# The Jrecan Device: Preclinical Data of a Novel Thrombectomy Device in Acute Thromboembolism Model of Beagle Dogs

**DOI:** 10.3389/fneur.2022.858670

**Published:** 2022-03-28

**Authors:** Chen Li, Ji Zhou, Yi-Qun Zhang, Jin Lv, Ying-Ying Zhang, Han-Cheng Qiu, Ao-Fei Liu, Wei-Jian Jiang

**Affiliations:** ^1^Department of Vascular Neurosurgery, PLA Rocket Force Characteristic Medical Center, Beijing, China; ^2^Department of Neurosurgery, Tiantan Hospital, Beijing, China

**Keywords:** stroke, thrombectomy, stent, animal model, endothelium

## Abstract

**Objective:**

The aim of this study is to investigate the safety and efficiency of a Jrecan^®^ flow restoration system, a novel thrombectomy device, in an arterial thromboembolic occlusion model of Beagle dogs.

**Methods:**

A total of 12 Beagle dogs with acute thromboembolism were randomized to receive mechanical thrombectomy with either Jrecan^®^ flow restoration device or Trevo^TM^ PROVUE Device (2:1). The efficacy and safety of the two devices, including recanalization rate, the presence of distal embolism, vasospasm, vessel perforation, and vessel injuries were evaluated through DSA and microscopic examination.

**Result:**

A 100% recanalization rate (mTICI 2b/3) was achieved in both groups. Endothelial and subendothelial injuries occurred in all target vessels. Focal disruption of internal elastic lamina was observed in 4 cases. The mean vessel injury score of the Jrecan^®^ group was 1.16 ± 0.48, significantly lower than that of the Trevo^TM^ group (1.54 ± 0.8) (*P* < 0.001).

**Conclusion:**

The Jrecan^®^ and Trevo^TM^ devices demonstrated an equally high recanalization rate in Beagle dogs with acute thromboembolism. However, histological findings revealed that the Jrecan^®^ stent seemed to be safer than the Trevo^TM^ device during clot retrieval, which might be related to a more appropriate radial force provided by the Jrecan^®^ stent that resulted from its wider cell design.

## Introduction

Acute ischemic stroke (AIS) is the leading cause of disability and mortality worldwide. Mechanical thrombectomy is widely accepted as an important and effective treatment for AIS (1–5). However, mechanical thrombectomy is associated with many procedural (4–29%) or postoperative complications (0.6–9.3%) (6), which both neurointerventionists and stroke specialists are concerned about. The thrombectomy device plays a key role in these complications. Some complications are device-related, which occur with the primary thrombectomy device itself. As seen on angiography, there may be gross morphologic changes, such as arterial perforation (0.9–4.9%) (7–9), vasospasm (3.9–9.3%) (7, 10, 11), and kinking or dissection of the target arteries (0.6–3.9%) (7–10). On microscopic examination, there may be severe disruption of the intima. Other complications, such as distal embolization, are not directly device-induced but might be reduced with a better-designed thrombectomy device. Therefore, it is important to improve the devices to enhance their clot retrieval safety without sacrificing efficiency.

In this study, we report the evaluation of the safety and efficacy of Jrecan flow restoration device (Rui Kang Tong, Changsha, China), a new thrombectomy device designed to reduce complication risk, by comparison with the Trevo PROVUE Device in a canine model. We, therefore, tested the hypothesis that some thrombectomy safety problems could be resolved with a better stent retriever.

## Methods

All procedures were conducted according to international guidelines at the Animal Experimental Center, Fuwai Hospital, Chinese Academy of Medical Sciences, and were approved by the Institutional Animal Ethics Committee [0077-3-14-HX(X)].

### Device Description

**Figure 1:**
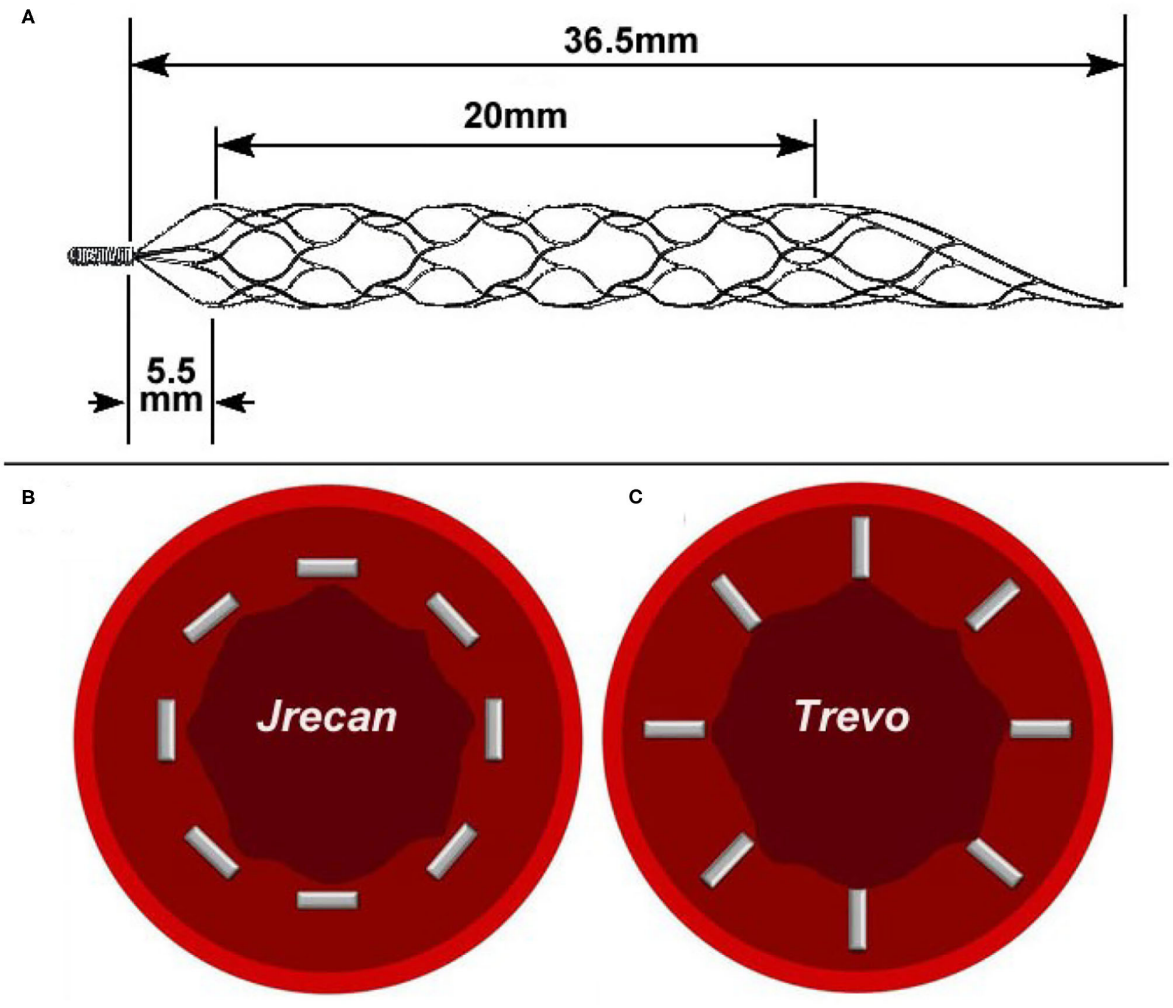
Physical picture and trabecular structure diagram of two devices. **(A)** The Jrecan^®^ device has an optimized grid design with a large mesh area (11.7 mm^2^) and it is designed to optimize the radial support force (0.120 N/mm). The area of the mesh at the distal end of the device is gradually reduced and formed into a conical shape. **(B)** The horizontal trabecular structure of Jrecan device. **(C)** The vertical trabecular design of Trevo device.

The Jrecan flow restoration device is a self-expanding laser-cut nitinol stent-based clot retriever. Different from the Trevo or Solitaire device, the Jrecan has wider cells (11.7 mm^2^) to provide the appropriate radial force, which results in less endothelial injury and proper clot fixation (Figure 1). It has a closed-ended capture basket at the distal portion designed to reduce distal embolization during thrombectomy. The distal end has a 4–7.5 mm soft atraumatic radiopaque tip for safe deployment and easy fluoroscopic visualization. The proximal 10−20 mm end is tapered to facilitate resheathing and has a radiopaque marker. The device is available in diameters of 2.5, 4, and 6 mm. The 2.5 mm device (length 15 mm) is recommended for usage in vessel diameters ranging from 1.5–2.5 mm. The 4 and 6 mm devices (length, 15, 20, 25, or 30 mm) are deployed in recommended vessel diameters of 2–4 mm and 3–5.5 mm, respectively. The device is provided with its own microcatheter, which is 1.9F, 2.1F, and 2.8F for the three diameter sizes. In this study, the 4 × 20 mm Jrecan stent was used.

### Thrombus Preparation

Autologous whole blood drawn 24 h before the procedure was used to create a thrombus. After gently mixing 10 ml autologous blood with 1 ml fibrinogen (0.1 g/ml, Borsi Science and Technology B1016, Beijing) and 400 IU bovine thrombin (Borsi Science and Technology B1019, Beijing) in a 50 ml syringe for 10 s, the mixture was injected into a polyvinyl chloride tubing (5 mm inner diameter) and incubated for 2 h at room temperature. The generated thrombus was then stored at 4°C in a freezer. Before application, the thrombus was drained and rinsed with physiologic saline solution and then cut into equal lengths of 20 mm. The final size of each thrombus was 5 mm in diameter and 20 mm in length.

### Animal Model

Twelve adult male Beagle dogs, weighing 11–15 kg, were used in this study. Sedation was induced by propofol (5 mg/kg), and general anesthesia was maintained by inhalation of 2% isoflurane and 100% oxygen. Short 6F sheaths (Cordis) were introduced into the bilateral femoral arteries and continuously flushed with heparinized saline (1 IU/mL). No additional heparin or other thrombolytic drugs were used during the procedure. After a 6F guiding catheter (Envoy; Cordis Neurovascular, Miami, FL, USA) was placed in the proximal right common carotid artery (RCCA), an angiogram was performed to identify the external carotid artery (ECA) and internal maxillary artery (IMA) suitable for modeling and thrombectomy. To ensure stable thrombus application without distal migration, a reticulated ball-shaped blocking device (5 mm in diameter) (Hunan Ruikang Technologies Co., Ltd., China) was used. The midpoint of the blocking device was placed in the origin of the ECA. Then 3 thrombi were injected through the guiding catheter into the RCCA to achieve occlusion proximal to the blocking device. A control angiogram was performed after 30 min to confirm the vessel occlusion. If spontaneous revascularization was observed, which in most cases resulted from clot autolysis rather than clot migration, another 2 thrombi were injected until successful embolization was confirmed by angiography. The reticulated ball-shaped blocking device was then carefully removed over the microcatheter and a final angiography was performed to document occlusion. The occlusion was maintained for 1 h (±10 min) before attempting thrombectomy.

### Randomization

The randomization was done of 12 dogs in a 2:1 ratio of Jrecan^®^ to Trevo^TM^ device. Treatment allocation was determined according to a computer-generated random number list prepared by a third party, with assignments concealed in sequentially numbered, opaque, and sealed envelopes.

### Revascularization Procedure

A 2.3F microcatheter (Prowler Select Plus, Cordis) was navigated over a 0.014 micro guidewire (Synchro, Stryker) across the occlusive thrombus. A Jrecan device (4 × 20) or a Trevo^TM^ device (4 × 20) was delivered through the microcatheter and deployed across the clot under fluoroscopy. Angiographies were performed immediately and 5 min later to check blood flow restoration after stent deployment. Then the device and microcatheter were slowly pulled back into the guiding catheter, while aspiration was performed via a 25-ml syringe. We repeated the procedure until modified treatment in cerebral ischemia (mTICI) score ≥2b was achieved or a maximum of three thrombectomy passes were reached. The number of retrieval attempts was recorded (12). A post-thrombectomy angiogram was obtained at 1 h to document recanalization and complications including perforation, vasospasm, and distal embolization.

### Angiographic Evaluation

Angiographies immediately after the deployment of the Jrecan or Trevo device and the final recanalization state were reviewed to evaluate the efficacy and safety of the two devices. All images were analyzed by two experienced neuroradiologists with 5 and 10 years of experience, respectively. Both neuroradiologists were blinded to the devices used and the procedural process.

The mTICI score was used to assess recanalization as follows (13): mTICI 0, no perfusion; mTICI 1, antegrade reperfusion past the initial occlusion, but limited distal branch filling with little or slow distal reperfusion; mTICI 2a, antegrade reperfusion of less than half of the occluded target artery previously ischemic territory; mTICI 2b, antegrade reperfusion of more than half of the previously occluded target artery ischemic territory; mTICI 3, complete antegrade reperfusion of the previously occluded target artery ischemic territory, with the absence of visualized occlusion in all distal branches. Our subsequent thrombectomy was performed in the ECA, and the target vessel region we observed was the ECA and all its branches. Successful recanalization was defined as mTICI 2b or 3 in the right ECA.

Angiographic safety was evaluated by the presence of distal embolism, vasospasm, and vessel perforation in ECA. Distal embolism was defined as complete or partial occlusion of the distal vessel lumen, confirmed by the final angiography. Vasospasm was graded using a standard scale (14): none/mild = vessel diameter reduction of 0–25%, moderate 26–50%, and severe >50%.

### Histological Examination

After the thrombectomy procedure, all dogs were euthanized with 10% potassium chloride (2 ml/kg, IV). The target vessels were dissected and sent to an independent laboratory for histopathological examination. The tissues were embedded in paraffin and cut into five 1-cm-thick coronal blocks from distal to proximal with regard to arterial blood flow. Then five serial 4-μm-thick sections were cut from every block again in a distal-to-proximal direction (25 sections per dog) and stained with hematoxylin–eosin and elastin stain. On microscopic inspection, the degree of vascular injury was graded on a 0–5 scale: 0, no damage; (1) only endothelial damage; (2) subendothelial injury in the target vessel; (3) damage to the internal elastic lamina; (4) smooth muscle damage; and (5) damage to the external elastic lamina or even tunica adventitia. All samples were scored by an experienced pathologist blinded to the group assignment.

### Statistical Analysis

Data analysis was performed using SPSS version 20 (Chicago, IL, USA). Analysis was carried out using rank-sum test, independent sample *t*-test, and by Chi-square test as appropriate. A *P* < 0.05 was considered significant.

## Results

### Efficacy

**Figure 2:**
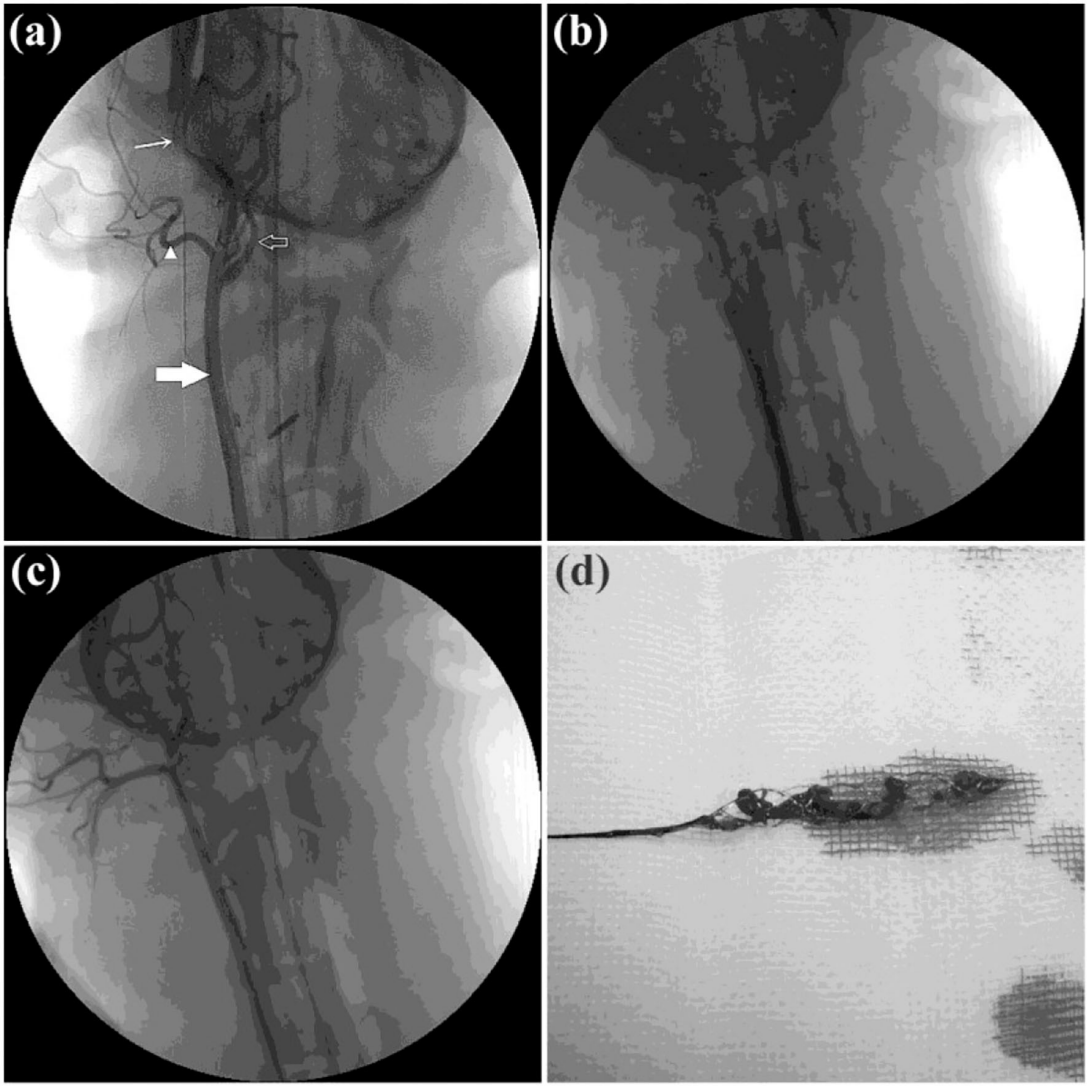
Angiographic results of the thrombectomy in beagle dogs. **(a)** Baseline carotid artery angiography data of the beagle dog. Common carotid artery is indicated by the thick white arrow. Posterior ear artery is indicated by the white triangle. Maxillary artery is indicated by the fine white arrow. Internal carotid artery is indicated by the white hollow arrow. **(b)** Acute dog external carotid artery thromboembolism modeling was created successfully. **(c)** DSA immediately after thrombectomy. TIMI/TICI 3 flow was achieved immediately after retrieval of the Jrecan device. **(d)** Thrombus after extraction.

Twelve RCCAs were successfully occluded by clot injection in all twelve Beagle dogs and were randomly allocated into the Jrecan^®^ group (*n* = 8) and the Trevo^TM^ group (*n* = 4). Successful recanalization was achieved in all dogs following clot retrieval, and there was no significant difference between the two groups. A total of 27 thrombectomy attempts were made, and the mean number of attempts taken was 2.1 ± 0.35 per vessel for the Jrecan^®^ device and 2.5 ± 0.58 for the Trevo^TM^ device (Table1) (Figure 2).

**Table 1:** Comparison of the safety and efficiency between Jrecan^®^ and Trevo^TM^ PROVUE.

	**Total**	**Jrecan^®^** **(*n* = 8)**	**Trevo^**TM**^ PROVUE (*n* = 4)**	***P*** **Value**
Gender	Male	Male	Male	
Weight (kg)	12.42 ± 0.66	12.28 ± 0.54	12.70 ± 0.86	
**mTICI**				
2b	50.0% (6/12)	50.0% (4/8)	50.0% (2/4)	
3	50.0% (6/12)	50.0% (4/8)	50.0% (2/4)	
≥2b (2b/3)	100.0% (12/12)	100.0% (8/8)	100.0% (4/4)	
**Number of attempts taken**	2.25 ± 0.45	2.1 ± 0.35	2.5 ± 0.58	
Number of slices	300	200	100	
Vascular injury score	1.29 ± 0.63	1.16 ± 0.48	1.54 ± 0.8	0.001^**^
**Acceptable injury**				
Grade 0	3.3% (10/300)	2.5% (5/200)	5.0% (5/100)	0.000^**^
Grade 1	69.7% (209/300)	80.5% (161/200)	48.0% (48/100)	
Grade 2	22.3% (67/300)	15.0% (30/200)	37.0% (37/100)	
**Unacceptable injury**				
Grade 3	4.0% (12/300)	2.0% (4/200)	8.0% (8/100)	
Grade 4	0.7% (2/300)	0.0% (0/200)	2.0% (2/100)	
**Vasospasm**				
No	33.3% (4/12)	50.0% (4/8)	0.0% (0/4)	>0.05
Mild	41.7% (5/12)	12.5% (1/8)	100% (4/4)	
Moderate	16.7% (2/12)	25.0% (2/8)	0.0% (0/4)	
Severe	8.3% (1/12)	12.5% (1/8)	0.0% (0/4)	
Distal embolization	16.7% (2/12)	12.5% (1/8)	25% (1/4)	1.000
Vascular perforation	0.0% (0/12)	0.0% (0/8)	0.0% (0/4)	
Dissection	0.0% (0/12)	0.0% (0/8)	0.0% (0/4)	
Rethrombosis	8.3% (1/12)	12.5% (1/8)	0.0% (0/4)	
Distal migration of the thrombus	50.0% (6/12)	50.0% (4/8)	50.0% (2/4)	

*mTICI (modified Treatment in Cerebral Ischemia): mTICI 0, no perfusion; mTICI 1, antegrade reperfusion past the initial occlusion, but limited distal branch filling with little or slow distal reperfusion; mTICI 2a, antegrade reperfusion of less than half of the occluded target artery previously ischemic territory; mTICI 2b, antegrade reperfusion of more than half of the previously occluded target artery ischemic territory; mTICI 3, complete antegrade reperfusion of the previously occluded target artery ischemic territory, with absence of visualized occlusion in all distal branches*.

*P < 0.05 and

** *P < 0.01*.

### Safety

#### Angiography

Vasospasm occurred in 4/8 (50%, 1 severe, 2 moderate, and 1 mild) cases of the Jrecan^®^ group and 4/4 (100%, all mild) of the Trevo^TM^ group. However, there was no significant difference (*P* > 0.05). Distal embolization was seen in 1/10 (10%) vessels of the Jrecan^®^ group and 1/7 (14.3%) vessels of the Trevo^TM^ group, but no significant difference was observed (*P* = 0.078). The treated vessels showed no angiographic evidence of vascular perforation or dissection (Table 1).

#### Histology

**Figure 3:**
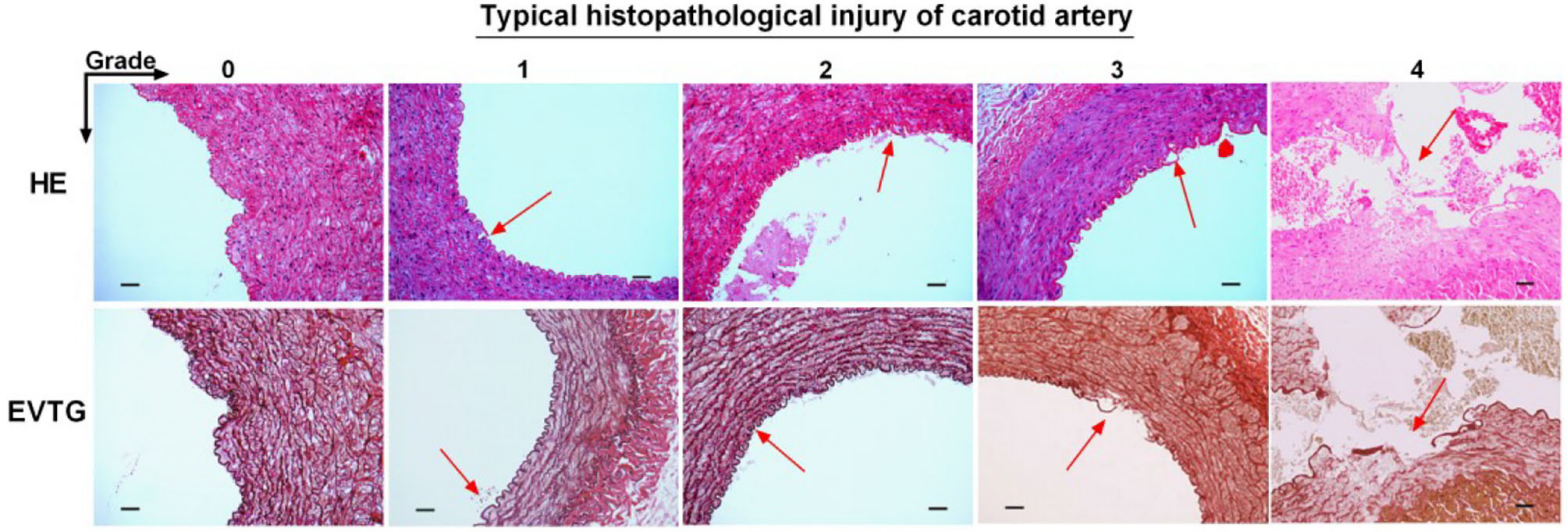
Histopathological injury of carotid artery. The tissues were embedded in paraffin and cut into five 1-cm-thick coronal blocks from distal to proximal with regard to arterial blood flow. Then five serial 4-μm-thick sections were cut from every block still in a distal-to-proximal direction (25 sections per dog) and stained with hematoxylin-eosin and Elastic Verhoeff's Van Gieson stain. The sections were examined under light microscopy (magnification 200x). The arrow represents the typical histopathological injury of carotid artery. Scale bar = 200 μm.

**Figure 4:**
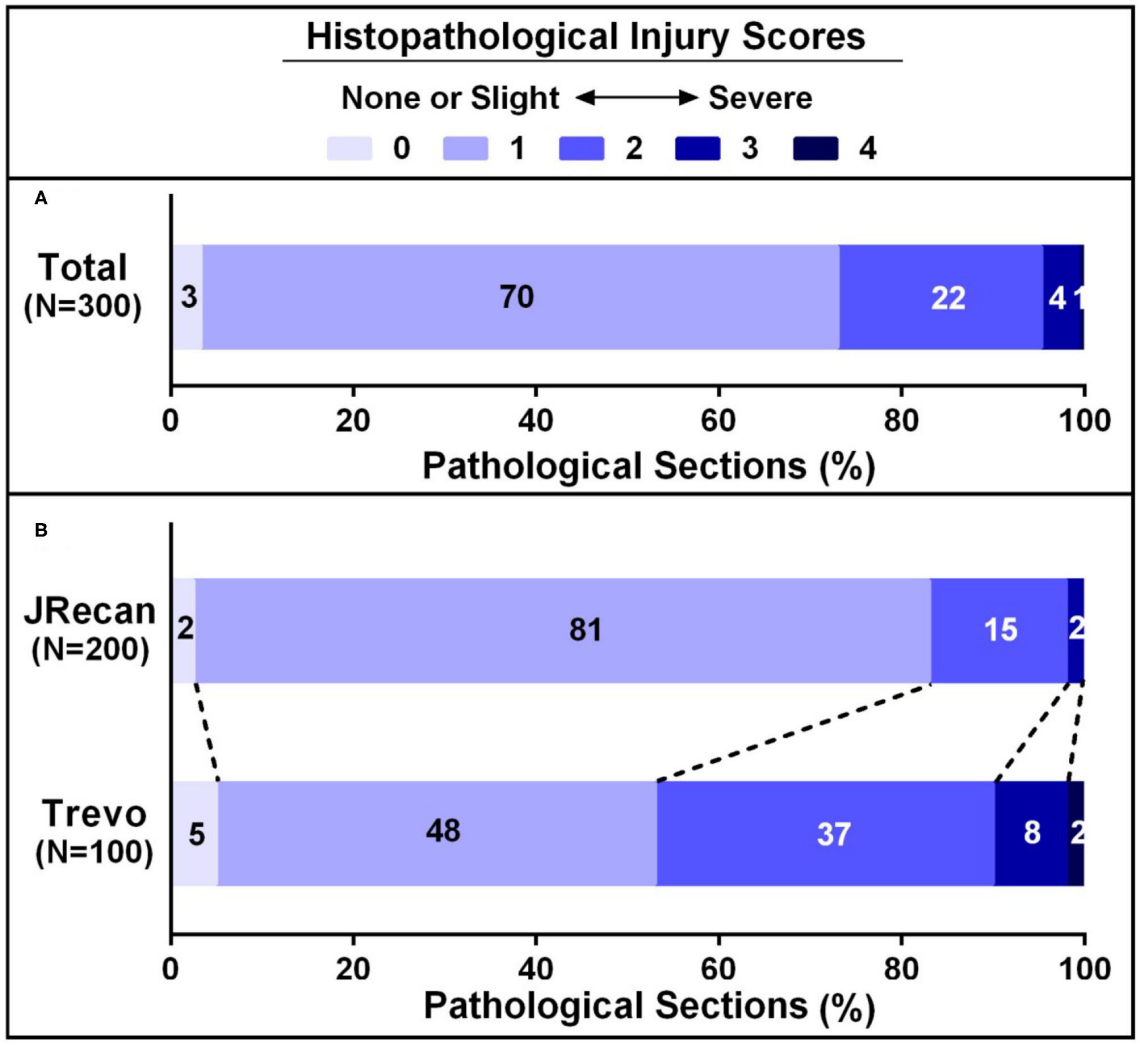
Histopathological injury scores of carotid arteries underwent Jrecan and Trevo thrombectomy. **(A)** Overall histopathological lesion score of carotid injury underwent Jrecan and Trevo thrombectomy. **(B)** Histopathological lesion score of carotid injury with respect to different thrombectomy devices. Data were presented as a percentage of the number of samples in each score to the total numbers of corresponding samples. Significance was determined by Wilcoxon rank-of-rank test.

Three 100 sections of target vessels, 200 in the Jrecan group and 100 in the Trevo group, were examined. Endothelial lesion was seen in all the vessels. Four cases of focal disruption of the internal elastic lamina and 1 case of smooth muscle injury were observed (Figures 3, 4). The mean vascular injury score of the Jrecan group was 1.16 ± 0.48 and the average score of the Trevo group was 1.54 ± 0.8 (*P* = 0.001). The Jrecan device caused significantly less damage to the vessel wall than the Trevo device.

## Discussion

This study confirmed that the newly developed Jrecan^®^ flow restoration device reduced blood vessel damage and effectively prevented the occurrence of distal embolization on the premise of ensuring the efficiency of thrombectomy.

The damage caused by the stent to the blood vessel wall has always been a great concern for neurologists and is an urgent problem to be solved during the process of clinical thrombectomy. Recently, the preclinical trial of Trevo system on a swine thrombo-occlusion model (15) reported that severe intima disruption occurred under light microscopy (LM). Instead, a study using the Solitaire system on a swine renal artery model showed that only minimal intimal thickening occurred during follow-up studies under a microscope (16) after mechanical thrombectomy. It is speculated that direct compression of the device on the carotid artery endothelium and the force exerted on the vessel wall during the withdrawal of the device along the axis were the main factors that caused endothelial cell damage. In this study, our experiment demonstrated a lower pathological impairment score on the blood vessel wall of the Jrecan device compared with the Trevo device, which might be attributed to the structural modifications of the Jrecan^®^ flow restoration device. The main innovation was the optimized radial support force (Jrecan: 0.120 vs. Trevo: 0.122, N/mm), which could reduce the damage to the blood vessel while not affecting the thrombectomy efficiency. Another modification of Jrecan device was the mesh design with a horizonal trabecular structure, different from the vertical trabecular design of mesh in Trevo device. Theoretically, the vertical trabeculae with a small contact area to the blood vessel wall could be better embedded in thrombus, but the cutting force on the vessel wall during thrombectomy might be greater, resulting in a damage to the larger vessel wall (Figure 3).

In addition, the present study showed that the Jrecan^®^ device obtained a comparable recanalization rate to the Trevo system and was an effective thrombectomy tool for the treatment of acute thrombus occlusion model in canines. With respect to the side branch affection or distal vessel embolism during thrombectomy, the Jrecan^®^ device also showed similar safety when compared with the Trevo system. These effects were closely related to the improvement of the mesh area at the distal end and the optimized grid design with a large mesh area (11.7 mm^2^), which was beneficial to avoid the small thrombus from falling off, to reduce the incidence of distal embolization, and to make it more excellent in bending performance and smoother in recycling during the thrombectomy process.

The major limitation is that this study only observed the acute phase of injury after thrombectomy and did not involve the long-term effects of these endothelial injuries. Whether the acute injury to the blood vessel wall caused by thrombectomy would cause long-term negative effects is still a question worthy of in-depth study. Previous studies showed that the vessel wall injury caused by thrombectomy could be the source of acute platelet adhesion and aggregation, leading to acute arterial re-occlusion and late intimal hyperplasia (12). One of them showed that mild intimal thickening occurred at day 30, and minimal to mild intimal thickening occurred at day 90 after thrombectomy, but no evidence of medial or adventitial proliferation was found. Although this thickening led to an ~5% luminal narrowing in three vessels and 1% luminal narrowing in one vessel, the stenosis had no significant effect on clinical and histopathologic changes (16). In addition, the present study is a preclinical animal research and therefore cannot be extrapolated directly to humans. Intracranial human arteries are more tender, tortuous, and have a different vessel wall structure from extracranial arteries. Thirdly, the cerebral arteries of most patients with acute ischemic stroke are atherosclerotic, which is not the case in our study. Finally, we only tested the performance of the stent retriever for soft thrombus, whereas firm clot was more frequently encountered in clinical situations. Therefore, the effectiveness and safety of the Jrecan device need to be further evaluated in clinical trials.

## Conclusion

In this animal experiment, both the Jrecan and the Trevo stent could effectively restore the blood flow of the occluded vessels and there was no significant difference. For device safety, the main injury caused by both thrombectomy devices for the blood vessels was intima injury, which is clinically acceptable. However, the Jrecan^®^ flow restoration device seemed to induce less vascular damage compared with the Trevo^TM^ PROVUE Device, which may give credit to its more suitable radial force provided by the wider cell design.

## Data Availability Statement

The raw data supporting the conclusions of this article will be made available by the authors, without undue reservation.

## Ethics Statement

The animal study was reviewed and approved by Institutional Animal Ethics Committee of Animal Experimental Center, Fuwai Hospital, Chinese Academy of Medical Sciences [0077-3-14-HX(X)].

## Author Contributions

CL had full access to all the data in the study and takes responsibility for the integrity of the data, prepared all tables and figures in the manuscript, and prepared the manuscript draft with important intellectual input from JZ, Y-QZ, JL, Y-YZ, and H-CQ. W-JJ designed and conducted the study. A-FL and H-CQ helped conduct the study, provided input, and helped with patient recruitment and consent. All authors listed have made substantial, direct, and intellectual contribution to the work and approved it for publication.

## Funding

This work was funded by National Key Basic Research Program of China (973 program) (grant no. 2013CB733805) and National Natural Science Foundation of China (grant nos. 81271536, 81070925, 81371540, 81101033, and 81871464).

## Conflict of Interest

The authors declare that the research was conducted in the absence of any commercial or financial relationships that could be construed as a potential conflict of interest.

## Publisher's Note

All claims expressed in this article are solely those of the authors and do not necessarily represent those of their affiliated organizations, or those of the publisher, the editors and the reviewers. Any product that may be evaluated in this article, or claim that may be made by its manufacturer, is not guaranteed or endorsed by the publisher.

## Abbreviations

RCCA: right common carotid artery
ECA: external carotid artery
IMA: internal maxillary artery
HE: hematoxylin-eosin staining
mTICI: modified Treatment in Cerebral Ischemia.
